# Self-management of diabetes in Sub-Saharan Africa: a systematic review

**DOI:** 10.1186/s12889-018-6050-0

**Published:** 2018-09-29

**Authors:** Victor Stephani, Daniel Opoku, David Beran

**Affiliations:** 1 Department of Health Care Management, Technical University of Berlin, Berlin, Germany; 2 Division of Tropical and Humanitarian Medicine, University of Geneva and Geneva University Hospitals, Genève, Switzerland

## Abstract

**Background:**

The prevalence of diabetes in sub-Saharan Africa has increased rapidly over the last years. Self-management is a key element for the proper management, but strategies are currently lacking in this context. This systematic review aims to describe the level of self-management among persons living with type 2 diabetes mellitus in sub-Saharan Africa.

**Method:**

Relevant databases including PubMed, Web of Science and Google Scholar were searched up to September 2016. Studies reporting self-management behavior of people with type 2 diabetes mellitus and living in sub-Saharan Africa were included.

**Results:**

A total of 550 abstracts and 109 full-text articles were assessed. Forty-three studies, mainly observational, met the inclusion criteria. The studies showed that patients rarely self-monitored their glucose levels, had low frequency/duration of physical activity, moderately adhered to recommended dietary and medication behavior, had poor level of knowledge regarding diabetes related complications and sought traditional or herbal medicines beside of their biomedical treatment. The analysis also revealed a lack of studies on psychosocial aspects.

**Conclusion:**

Except for the psychosocial area, there is a good amount of recent studies on self-management behavior of type 2 diabetes mellitus sub-Saharan Africa. These studies indicate that self-management in sub-Saharan Africa is poor and therefore a serious threat to the health of individuals and the health systems capacity.

**Electronic supplementary material:**

The online version of this article (10.1186/s12889-018-6050-0) contains supplementary material, which is available to authorized users.

## Background

Although the true burden of diabetes in sub-Saharan Africa (SSA) is unknown, it is recognized as a serious challenge to health systems [1, 2]. Current prevalence-estimates range between 2.1 and 6.0%, and the number of people suffering from the disease is likely to double within the next 25 years [3]. In order to reduce the burden posed to health systems and affected individuals, patients with diabetes need to adopt certain self-management behaviors. The American Diabetes Association (ADA) has therefore defined a list of essential self-care behaviors, which have been found to be positively correlated to good glycemic control and a reduction of complications [4, 5]. Diabetes Self-Management Education (DSME) is critical for informing patients about these essential self-care behaviors. Currently, DSME in most African countries is limited in scope, content and consistency and it is not clear how patients from SSA manage their diabetes [6–8]. Therefore, the aim of this systematic review is to assess the status of self-management of people with diabetes in SSA, and to analyze to what extent they follow the recommended self-management behavior.

## Method

### Search strategy and screening procedure

A preliminary search was performed in order to find appropriate terms. The final search strategy was discussed among the authors (VS and DO). Search term categories belonged to: “Diabetes”, “Sub-Saharan Africa” and “Self-management”. Databases included in the search were PubMed, Web of Science and Google Scholar. In addition, reference lists of screened studies were checked. An example of the performed search and the key words used is provided in Additional file 1.

The search-strategy yielded 741 publications (MEDLINE 436, Web of Science 232, Google Scholar 50). After removal of duplicates, 550 studies remained. VS and DO reviewed titles, keywords and abstracts independently and discussed the eligibility for full-text inclusion.

After discussing results and resolving disagreements, full texts of the remaining 109 publications were screened for eligibility. The overlapping rate of included and excluded studies was 87% between both authors. Disagreements were discussed and resolved by consensus, resulting in forty-three articles included in this review.

### Inclusion criteria

Studies were included for this review if they met the following inclusion criteria:

- They took place in at least one country from sub-Saharan Africa, as defined by the World Bank [9]
- Participants were people living with type 2 diabetes mellitus (which accounts for 90% of all diabetes cases in SSA [10])
- The study analyzed self-management behavior of type 2 diabetes patients as defined by the American Diabetes Association (ADA) as described in Table 1. If a study analyzed both, type 1 and type 2 diabetes, it was only included if the outcome measures (or self-management behavior) for patients with type 2 diabetes were presented separately
- Published anytime before September 2016 (with no limit concerning the start date)
- The study was published in English or German

**Table 1:** Specification of categories and included outcomes used for the analysis of self-management as given by the ADA [5]

Category	Specification	Included Outcomes
Healthy eating	General awareness of its importance, awareness of importance of measuring and portioning meals, adherence to an eating plan	Eating behavior, knowledge on diet recommendations, presence of and adherence to a diet plan
Being active	General awareness, existence of and adherence to an activity plan (with information on frequency, intensity, time and type of activity), glucose checking before and after sports	Knowledge on activity recommendations, presence of and adherence to an activity plan
Monitoring	General awareness, conducting SMBG (including information on frequency), keeping record of results, ability to analyze results	Awareness of SMBG, Availability of a glucose meter at home, frequency of SMBG
Taking Medication	Awareness of the kind of prescribed medicine, adherence to the medication plan	Prescribed medication, medication adherence, awareness that medication needs to be taken throughout the life-time
Reducing Risks	Awareness of possible complications, tobacco consumption, regular doctor appointments, taking care of feet	Awareness of consequences of uncontrolled Diabetes, consultations of specialists, self-care behavior, cigarette intake
Psychosocial Aspects	Environmental, social, emotional burden of diabetes	Support by relatives, emotional and environmental aspects

Table 1 presents all self-management related outcome categories and specifies them according to the recommendations given by the ADA [11].

### Data extraction, analysis, and synthesis

Two data extraction templates (using Microsoft Excel) were developed to gather all data relevant for the analysis. One template was used for collecting characteristics of included studies (e.g. year of publication, country, number of participants, number of woman/man, age); study results and relevant information on self-management were collected in a second template. Qualitative and quantitative results were combined and summarized according to their specific area of self-management. Quantitative results were rounded to the nearest full percent and study-size-weighted arithmetic averages were calculated if eligible.

Risk of bias was assessed and information about the quality of the included studies were derived from the text using quality-assessment tools for cross-sectional studies [12], pre-post studies [13] and randomized controlled trials [14]. Additional file 2 contains the full details of a PRISMA checklist for this review and the full risk assessment of the included studies can be found in the Additional files 3, 4, 5.

## Results

**Fig. 1:**
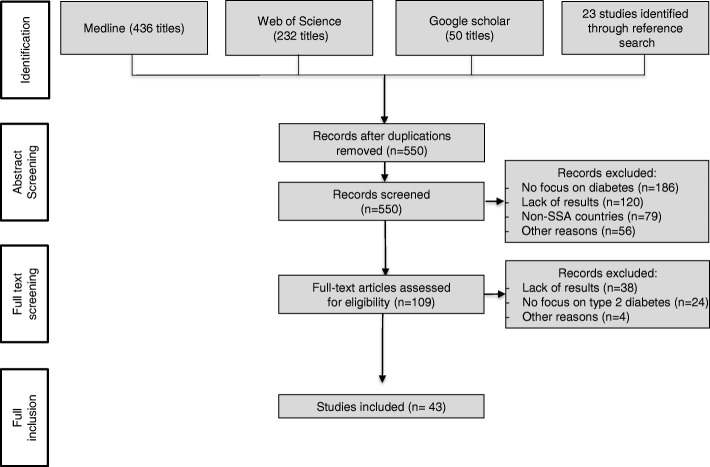
Literature screening process

The final analysis included forty-three studies. Figure 1 illustrates the literature search and selection process. Common reasons for exclusion were: lack of results, reports from non-SSA countries, or focus on other diseases than type 2 diabetes mellitus. Publication dates were between 2002 and 2016. The majority of studies (*n* = 33) were published after 2010.

### Description of included studies

Study characteristics such as the year of publication, sample size, study design and the measured outcome parameters of the forty-three included studies are summarized in Table 2. Most studies took place in Nigeria (*n* = 13) and South-Africa (*n* = 11), followed by Ghana (*n* = 6), Uganda (*n* = 4), Ethiopia (n = 3), Cameroon (*n* = 2), Tanzania, Kenya, Sudan, Zimbabwe (n = 1 each). Thirty-five studies were observational (mostly cross-sectional, only one longitudinal study [15]), while six studies were experimental (two studies described the same intervention [16, 17]). 8281 participants with type 2 diabetes were included with an average age above 50 years, and out of which 4676 were women (3 studies did not indicate how many men or women were included). People had been living with their diabetes on average over 5 years. Most studies dealt with the self-management area of medication (*n* = 26), followed by the assessment of nutritional intake and the engagement in physical activity (*n* = 21 and *n* = 20). Fifteen studies were about risk reduction and self-monitoring of blood glucose, respectively. Only three studies considered psychosocial aspects of people with diabetes.

**Table 2:** Characteristics of included studies

	Author	Year	Country	Study Type	Sample characteristics				Reported outcomes					
					Sample size	Male	Female	Average age	Healthy eating	Being active	Monitoring	Medication	Risk Reduction	Psychosocial
Observational studies	Awah [57]	2008	Cameroon	cross-sectional	20	11	9	62.5				x		
	Awah [15]	2009	Cameroon	cross-sectional	65	30	35	–						
	Kassahun [43]	2016	Ethiopia	cross-sectional	309	189	120	50			x	x		
	Sorato [31]	2016	Ethiopia	cross-sectional	194	95	99	50.3	x	x	x	x		
	Wabe [42]	2011	Ethiopia	cross-sectional	384	186	161	48.3			x	x	x	
	Bruce [50]	2015	Ghana	cross-sectional	200	95	105	–				x	x	
	de-Graft Aikins [23]	2014	Ghana	cross-sectional	20	2	18	60	x		x	x	x	
	Doherty [27]	2014	Ghana	cross-sectional	30	10	20	48.7	x					
	Mogre [30]	2016	Ghana	cross-sectional	222	74	148	48.4	x	x				
	Obirikorang [54]	2016	Ghana	cross-sectional	630	243	387	55.2					x	
	Obirikorang [26]	2016	Ghana	cross-sectional	543	232	311	51.1	x	x			x	
	Matheka [44]	2013	Kenya	cross-sectional	187	–	–	–			x			
	Adibe [38]	2011	Nigeria	cross-sectional	314	136	178	43		x			x	
	Adisa [24]	2009	Nigeria	cross-sectional	121	60	61	–	x	x	x	x		
	Adisa [29]	2011	Nigeria	cross-sectional	114	51	63	61.3	x	x	x	x		
	Awotibede [37]	2016	Nigeria	cross-sectional	299	105	194	51.9		x				
	Ezuruike [48]	2016	Nigeria	cross-sectional	112	43	69	46				x		
	Iwuala [47]	2015	Nigeria	cross-sectional	100	38	62	59.9			x			
	Jackson [51]	2015	Nigeria	cross-sectional	303	171	132	54.5				x		
	Ogbera [40]	2011	Nigeria	cross-sectional	150	50	100	69.9		x	x	x		x
	Onakpoya [49]	2010	Nigeria	cross-sectional	83	32	51	57.5				x	x	
	Oyetunde [52]	2014	Nigeria	cross-sectional	102	35	67	59.6				x		
	Yusuff [46]	2008	Nigeria	cross-sectional	200	110	90	–				x	x	
	Jackson [36]	2014	Nigeria	cross-sectional	303	132	171	50		x	x	x	x	
	Adeniyi [22]	2015	South Africa	cross-sectional	17	6	11	58.5	x	x		x	x	x
	Haque [53]	2005	South Africa	cross-sectional	–	–	–	–				x		
	Matwa [55]	2003	South Africa	cross-sectional	15	5	10	61.4					x	
	Mendenhall [56]	2015	South Africa	cross-sectional	27	–	27	59				x		
	Nthangeni [33]	2001	South Africa	cross-sectional	288	133	155	62	x			x		
	Okonta [39]	2014	South Africa	cross-sectional	217	–	–	51	x	x				
	Steyl [35]	2014	South Africa	cross-sectional	26	11	15	58.9	x	x		x		
	Abdelgadir [45]	2006	Sudan	cross-sectional	193	95	98	50			x			
	Kamuhabwa [32]	2014	Tanzania	cross-sectional	469	171	298	54.9	x	x	x	x		
	Hijelm [34]	2008	Uganda	cross-sectional	25	10	15	–	x	x		x	x	
	Mayega [28]	2014	Uganda	cross-sectional	96	48	48	47.5	x	x				
	Nielsen [41]	2016	Uganda	cross-sectional	10	6	4	65.6	x	x	x			
	Hijelm [25]	2010	Zimbabwe	cross-sectional	21	10	11	48	x	x	x		x	
Experimental studies	Awodele [21]	2015	Nigeria	pre-post, quasi-experimental	152	47	105	65				x		
	Baumann [18]	2015	Uganda	pre-post, quasi-experimental	25	7	18	53	x	x		x		
	Mash [20]	2014	South Africa	RCTs	1570	411	1158	56.4	x	x		x	x	
	Muchiri [16]	2015	South Africa	RCTs	41	5	36	59.4	x					
	Muchiri [17]	2015							x					
	van der Does [19]	2013	South Africa	RCTs	84	68	16	51.6	x	x		x	x	x

All experimental studies tested various forms of DSME programs, with either a pre-post design [18, 19], or a control group [16, 17, 20] study-design. One intervention was done by counselling and educating the patients on medication adherence at the beginning of the study [21]. In another study [18] patients attended a one-day education program. Two studies tested the impact of 4 one-hour group education sessions about the importance of nutrition, physical activity, adherence to medication and risk reduction [19, 20]. A more comprehensive intervention tested the outcome of weekly group education sessions on nutritional aspects, combined with monthly follow up sessions plus education in vegetable gardening [16, 17].

### Study results on self-management

#### Healthy eating

Twenty-one studies included information on healthy eating self-care behaviors. Participants understood that unhealthy eating is a dominant cause of diabetes [16, 22, 23] and that it is important to take aspects such as the sugar-, salt- or fat-level of consumed food into consideration [19, 22, 24–26]. However, misconceptions and gaps of knowledge were present; particularly about the definition of high risk food [19], the sugar-level of food [24, 27] and the underlying diabetes related metabolic mechanisms [24]. As found in one study, respondents did not know the proportion of food they were allowed to eat [24]. And another study showed that mostly men talked about regular meals, while most women did not [28]. ‘Positive dietary behavior changes’ because of their diabetes were reported by 33% of Nigerian [29], 51% of Ghanaian [30] and most of South African [16] participants. Regarding the adherence to a certain diet plan, 60% [31], 70% [32] and 87% [33] stated that they ‘followed an eating plan’.

Four experimental studies assessed the impact of counseling sessions on the adherence to diet plans. Two interventions assessed the impact of four one-hour group education sessions on nutritional aspects: One increased the level of adherence significantly from 4.8 to 5.9 days per week [19] and one decreased the adherence non-significantly from 4.8 to 4.6 days per week [20]. The third intervention, which combined weekly group educational sessions on nutritional aspects with monthly follow up sessions and education in vegetable gardening, significantly reduced the intake of energy and starchy food [17]. The fourth intervention, which consisted of weekly contacts among the patients over a period of four months, was found to improve the healthy eating habit of patients significantly from 11.5 points to 22.4 points (out of 25 total points on the ‘Diabetes Self-Management Assessment and Reporting Tool’) [18].

#### Being active

Seventeen observational studies assessed physical activity behaviors and three interventional studies tested the impact of group educational programs.

The majority of participants in six studies were aware of the importance of being active and of doing regular aerobic exercises (such as brisk walking or climbing staircases) as part of their non-medical treatment [22, 25, 34–37]. However, respondents in three studies showed that a majority did not understand the relevance of physical activity as part of their glycemic control and therefore revealed gaps in knowledge on recommended type, frequency and duration of physical activity [24, 38, 39]. In addition, men and women were not always equally well-informed [34].

No study mentioned that participants had an activity plan or kept records of type, frequency, time and intensity of all exercises, or did glucose checking before and after doing sports.

Five observational studies indicated a low engagement in practicing exercises: 29% mentioned to ‘practice exercise’ [29], and only 25% [19], 27% [37], 33% [32] and 46% [40] said they were engaged in exercises on a regular basis. The most common type of exercise among participants was brisk walking [26, 37].

Less than half of the people who were engaged in regular exercises did their exercise daily [26] and only 39% at least in 30 min of duration [37]. In one study [31], 50.5% of respondents from Ethiopia reported to be engaged in at least 30 min of physical activity for total of ≥3 days per week.

Interventions with frequent group education sessions had mixed results based on the studies identified. One study found a significant increase in physical activity from 3 to 4.5 days per week [19], one found a non-significant increase from 4.1 to 4.5 [18], and one found a non-significant decrease from 4 to 3.9 days per week [20].

#### Monitoring

**Fig. 2:**
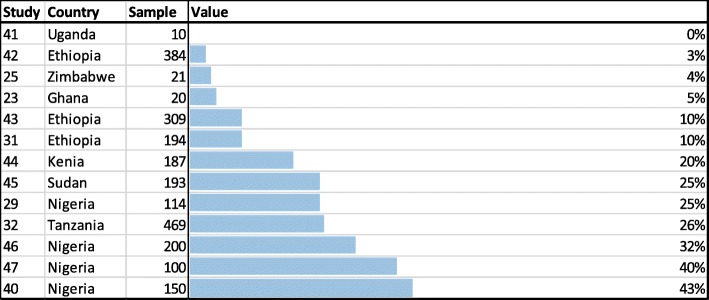
Percentage of people who are able to self-monitor their blood-glucose level at home

Fifteen observational studies reported on patients’ behavior regarding monitoring of blood glucose. The vast majority of respondents from Nigeria [24] and Zimbabwe [25] reported to not be aware of SMBG. Thirteen studies observed how many of the study participants had the possibility to self-monitor their blood glucose level and had access to a glucometer at home (Fig. 2). The results indicate a very low degree of SMBG, ranging from a study from Uganda, where none of the patients had access to a glucose meter at home [41] to one study from Nigeria with 43% of all patients doing glucose testing at home [40]. On average only 15% of all patients were able to test his or her blood glucose level at home [23, 25, 29, 31, 32, 40–47].

Most patients, who had access to a glucometer at home, checked their glucose level only once a month or at no regular interval [21, 45, 47]. Only 1% [21] and 2% [45] of respondents measured their glucose level on a daily basis. One study mentioned that women did SMBG more regularly than men [47]. Another study reported that half of those people who performed SMBG, also kept records of their results [40]. Most importantly, no study reported patients’ ability to analyze test results and whether they know what to do if their glucose numbers are off target.

#### Medication

Twenty-three observational and three experimental studies included information on peoples’ awareness and adherence to prescribed medication. The most common type of medication prescribed were oral hypoglycemic agents (*OHA*): On average, 86% were on OHA alone, while 7% were on a combination of OHA and Insulin and the remaining 7% were on Insulin alone [29, 31–33, 40, 42, 46, 48, 49]. The fact that diabetes drugs need to be taken throughout the life-time was known by the majority of patients in Nigeria [24, 29, 36] and Uganda [34].

**Fig. 3:**
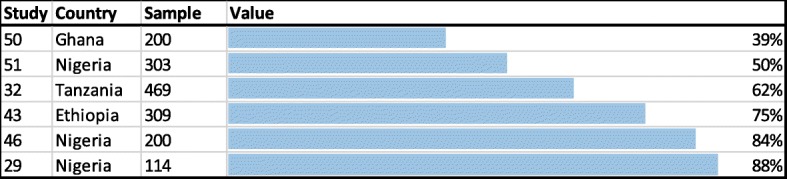
‘Morisky Medication Adherence Scale’ results showing the percentage of people with a moderate medication adherence (> 75% of adherence)

Six observational studies assessed patients’ medication adherence by using the Morisky Medication Adherence Scale (MMAS). It entails (8 or 4, depending on the MMAS-version) questions about the self-reported medical adherence. A perfect medication adherence is having a full score on the MMAS (meaning 8 or 4 points). Setting a cut-off point at 75% of the MMAS (indicating a moderate level of adherence), the adherence rate is on average 64% (see Fig. 3) [29, 32, 43, 46, 50, 51].

Six other studies asked for the non-adherence (instead of adherence) without utilizing a standardized questionnaire. The results ranged from 20% of people who had a “lack of adherence” [52], to 21% who stated that they “missed the medication” [42], to 35% who were classified as having a “poor adherence” [22], to half of all participants who reported that they “forget sometimes” to take their medication [24] and who do not “take the drugs on time” [40].

One study [53] asked the responsible diabetes doctors about their perception on patients’ adherence to prescribed medication. They concluded that the majority of all patients are non-compliant with the pharmacotherapy.

All three experimental studies improved medication adherence. A one-day education program in combination with weekly contacts among participants improved the frequency of ‘missed medication’ from 1.9 to 1.6 (1 never, 5 daily) [18], and the four one-hour group education programs about self-care behaviors improved the medication adherence from 6.3 to 6.5 days a week [19] and from 6.8 to 6.9 [20] days a week. However, all of these improvements resulted to be non-significant.

#### Risk reduction

Thirteen observational studies and two interventional studies dealt with risk reduction. Participants had various levels of knowledge about general consequences and complications of uncontrolled diabetes. All respondents from Ghana attributed complications to medical non-adherence [23] and most patients from a South African study [22] connected their already developed complications (e.g. foot problems, sexual dysfunction) to uncontrolled diabetes. However, only few participants were aware of the specific complications that could develop: the most frequently named complications were foot ulcers (on average named by 45%) and retinopathy (on average 36%) [42, 46, 50, 54, 55]. Other complications named were neuropathy (31%), sexual dysfunction (26%) [50, 54], or nephropathy (18%) [42, 50, 54]. The prevalence of cigarette smoking, which contributes to developing complications, appeared to be not very present and accounted on average for only 10% of all participants [18, 20, 31–33, 36, 43, 50, 54].

Having regular appointments at medical specialists (e.g. eye-doctor or dentist) is an important aspect of risk reduction. 77% of patients in one Nigerian study knew that they should go to the doctor when they have changes in their eyesight [38]. In another study 29% stated that they had previous dilated eye examinations [48]. On average, 80% [36, 38] of participants knew that they should take care of their teeth. No study assessed the frequency of visits at medical specialists.

Proper foot care is also critical for the reduction of risks. Most Nigerian diabetes patients knew that they have to take extra care of their feet [36]. In Zimbabwe only half of one group had been informed about foot care, and only with a limited content [25]. There was also a men-women discrepancy in one Ugandan study: women were better informed on how they should take care of their feet then men [34]. In one South African study all respondents reported that they adhered to the recommended foot care [55]. Two studies looking at group education programs about self-care behaviors, improved the foot care of participants non-significantly from 5.5 to 5.7 days per week [20] and significantly from 4.5 to 5.8 days per week [19].

#### Psychosocial aspects

Only three observational studies reported about the psychosocial aspects of having diabetes.

One study mentioned that the majority of patients received support from their family [22]. Stress and insufficient sleep due to the diabetes appeared to be below 1% among South African patients [19] and another study revealed a moderate level of emotional distress [40]. However, no study on environmental or other social aspects of living with diabetes was identified.

#### Alternative medicine

Although not included in the ADA framework (Table 1), alternative medicine was seen as an important component in SSA for self-management: Eleven studies addressed the utilization of alternative medicine by study participants. This shows that the western based model of self-management fails to describe the entire self-management behavior of diabetes patients in SSA. 11% of South African patients sought traditional healers [56] and many respondents from Cameroon stated that they used traditional diagnostic tools, such as tasting their urine for glucose [15]. Herbal medicine was equally valued with biomedical therapy [57] and frequently used [25]. The use of herbal medicines as part of the diabetes treatment was on average 32% [21, 34, 46, 48]. For some participants, it was grounded on their negative feelings and dissatisfaction towards biomedicine [15] or the belief that diabetes is a supernatural problem caused by witchcraft or fate [23, 25, 55]. To others, the willingness to treat diabetes took them to a 'modern' health facility but the willingness to cure diabetes took them to a traditional healer [15, 33, 56].

## Discussion

### Main findings and recommendations

This is the first systematic review which analyzes the self-management behavior of people with diabetes in SSA. Studies which analyzed **nutritional** aspects (*n* = 20) revealed a moderate level of adherence to recommended diet plans, with adherence rates ranging from 33 to 87% [16, 29–33]. Moreover, patients demonstrated a basic understanding of the right eating habits [16, 19, 22–26], but also revealed several gaps in their knowledge (e.g. regarding the sugar-level of food) [19, 24, 28]. Those which analyzed **physical activity aspects** of self-management behavior (n = 20) found that most patients were aware of the importance of aerobic exercises [22, 25, 34–37]. However, adherence rates to exercise plans varied between 29 and 46% [19, 26, 29, 31, 32, 37, 40]. Studies with information on the **medication** (*n* = 26) showed that Medication-adherence, measured by the MMAS questionnaire, was on the average 64% [29, 32, 43, 46, 50, 51]. Other studies, which utilized other (non-MMAS) methods confirmed these moderate results [22, 24, 40, 42, 52]. **Risk reduction** was assessed by 15 studies. Patients connected complications to uncontrolled diabetes, but only few were aware of the specific complications that can be developed [22, 23, 42, 46, 50, 54, 55] and how they can be prevented [25, 34, 36]. There was no study assessing the frequency of visits at medical specialists (such as an eye doctor or dentist) and only one study mentioned that all patients adhered to the recommended foot care [55]. Only three studies reported on **psychosocial aspects**. They indicated that people with diabetes seem to have a very low emotional distress level [19, 22, 40]. Although not part of the ADA self-management guidlines the use of **herbal medicine** and traditional healers was frequently mentioned [21, 25, 34, 46, 56–58]. Lowest adherence rates were assessed for patient’s ability to **self-monitor their blood glucose**. On average, only 15% were able to test the blood glucose at home [23, 25, 29, 31, 32, 40–47] – and only very irregularly [19, 21, 45, 47]. Studies which tested **DSME programs** (*n* = 6) showed significant improvements for eating and activity habits [16, 18, 19], medication adherence [21] and risk reduction behavior [19]. Improvements were ascertained for the adherence to activity and medication plans [18–20] and risk reduction behavior [20], but without significance. Also without any significance, negative effects were shown in only one study for eating and activity behaviors [20].

This review is important because it shows that self-management of diabetes in SSA is insufficient. Particularly, the lack of physical activity, the inappropriate risk reduction knowledge and behavior, and the missing ability to self-monitor blood glucose are a serious threat to good glycemic control. Medication and nutritional adherence appeared to be better but are still sub-optimal. By comparing the results with results from other countries outside SSA, we observe a similar ‘ranking’: The three elements ‘physical activity’, ‘risk reduction’ and ‘SMBG’ are also the most critical parts of self-management outside SSA (adherence rates of 45–54%), while the adherence to medication and nutrition plans is better: outside SSA medication plans are followed by 87% (vs 64% in SSA). And diet plans are followed by 76% outside SSA (vs 72% in SSA) [59].

Second, the review revealed that the (western-based) ADA model of self-management fails to describe all self-care activities in SSA. One third of all patients sought alternative medicine beside of their biomedical therapy (in non-SSA countries this is done by 8% [59]). For many people it is therefore part of the self-management. Future research should focus on the (unknown) ingredients of herbal medicines and their interactions with other taken medicines, such as OHA.

Third, the provision of structured DSME programs in SSA is found to be effective. Most of the measured self-management behaviors, such as the adherence to medication or diet plans, were significantly improved by DSME programs. This supports the existing literature, which has proven that DSME is effective in non-SSA countries [60]. Therefore, we recommend to improve the current distribution of structured context-adapted DSME programs in SSA. Important factors, such as the low access to blood glucometers or the utilization of alternative medicines, need to be considered when conceptualizing these programs. Other factors, which have not been addressed in this review, need to be considered as well, e.g. the shortages of healthcare workers [61] or the lack of medicines [62]. Moreover, the implementation of structured DSME programs could be supported by technology. So called mobile health (mHealth) solutions, which have shown to be effective against non-communicable diseases [63], could be used to guide health professionals through the education process and to follow up with patients.

Last, our results showed that there is only very limited research on psychosocial aspects in SSA. In contrast to all other self-management factors, we identified only three studies on psychosocial aspects (e.g. 21 studies on nutritional behavior or 15 studies about SMBG). Therefore, future research should put a higher emphasis on the assessment of the psychosocial situation, because factors such as stress or the missing support by the family can have a highly negative impact on people with diabetes and are associated with non-adherence to medication regimen and other self-management behaviors [64].

### Limitations

An important limitation of this review is that it combines studies from 10 countries, which are culturally and economically diverse. The generalizability of the results is therefore problematic, because it was not always clear whether the individual study results were representative (see risk of bias assessment, additional files 3-5). The studies also differ in their objective, e.g. while some evaluated DSME programs, others measured the adherence to OHA. However, combining studies from various countries with heterogeneous objectives is not unusual for reviews on diabetes in SSA [65]. Furthermore, methods applied to measure outcome-parameters varied among included studies. One example is the medication adherence: in some studies people where simply asked whether they “missed medication” or “forget sometime” to take their medication, while other studies used the standardized MMAS scale. Moreover, the analysis considers only patients who have been diagnosed with diabetes. It is estimated that around two thirds of all people who suffer from diabetes in SSA remain undiagnosed [3]. Another limitation concerns the method used by all included studies: most of the measured outcomes were self-reported. The use of self-reported measures, such as the medication adherence may underestimate the non-adherence of patients [52]. Multiple methods may be required to detect those who report adherence but who may in fact be non-adherent.

## Conclusion

There is a good amount of recent studies on self-management behavior of type 2 diabetes in SSA. These studies indicate that self-management in SSA is poor and a serious threat to glycemic control. Particularly, self-monitoring of blood glucose, physical activity and risk reduction behavior are insufficient. More research on the psychosocial situation is needed. Future efforts and resource investments in public health systems need to strengthen the distribution of strucutred DSME programs which need to be adapted to the SSA-context.

## Additional files

Additional file 1: Search strategy used. (DOCX 12 kb)

Additional file 2: PRISMA checklist. (DOCX 26 kb)

Additional file 3: Risk assessment for cross-sectional studies. (DOCX 21 kb)

Additional file 4: Risk assessment for pre-post studies. (DOCX 14 kb)

Additional file 5: Risk assessment for RCTs. (DOCX 13 kb)

## Abbreviations

ADA: American Diabetes Association
DSME: Diabetes Self-Management Education
MMAS: Morisky Medication Adherence Scale
NCD: Non Communicable Diseases
OHA: Oral Hypoglycemic Agents
SMBG: Self-Monitoring of Blood Glucose
SSA: Sub Saharan Africa
