# Supplementary material for: Self-management of diabetes in Sub-Saharan Africa: a systematic review
Source: BMC Public Health. 2018 Sep 29;18:1148. doi: 10.1186/s12889-018-6050-0 (PMC6162903; doi:10.1186/s12889-018-6050-0)
Supplement: Supplementary file 1 — Search strategy used. (DOCX 12 kb) [file 12889_2018_6050_MOESM1_ESM.docx]

Additional file 1: Search strategy used

| *Africa OR "Sub-Saharan Africa" OR Angola OR Benin OR Botswana OR Burkina Faso OR Burundi OR Cameroon OR Central African Republic OR Chad OR Congo OR Comoros OR Cote d'Ivoire OR Democratic Republic of the Congo OR Equatorial Guinea OR Eritrea OR Ethiopia OR Gabon OR Gambia OR Ghana OR Guinea-Bissau OR Kenya OR Lesotho OR Liberia OR Madagascar OR Malawi OR Mali OR Mauritania OR Mozambique OR Namibia OR Niger OR Nigeria OR Republic of Congo OR Rwanda OR Sao Tome and Principe OR Senegal OR Sierra Leone OR Somalia OR South Africa OR Sudan OR Swaziland OR Tanzania OR Togo OR Uganda OR United Republic of Tanzania OR Zambia OR Zimbabwe* |
| --- |
| *AND Diabetes* |
| *AND "Self-Management" OR "self-care" OR "self-control" OR empowerment OR knowledge* |
