# Supplementary material for: Self-management of diabetes in Sub-Saharan Africa: a systematic review
Source: BMC Public Health. 2018 Sep 29;18:1148. doi: 10.1186/s12889-018-6050-0 (PMC6162903; doi:10.1186/s12889-018-6050-0)
Supplement: Supplementary file 3 — Risk assessment for cross-sectional studies. (DOCX 21 kb) [file 12889_2018_6050_MOESM3_ESM.docx]

**Quality assessment of included cross-sectional studies,**

Adapted from Guyatt GH, Sackett DL, and Cook DJ, Users’ guides to the medical literature. II. How to use an article about therapy or prevention. JAMA 1993; 270 (21): 2598-2601 and JAMA 1994; 271(1): 59-63

| **#** | **Screening Questions** |
| --- | --- |
| 1 | Did the study address a Clearly focused issue? |
| 2 | Did the authors use an appropriate method to answer their question? |
| 3 | Were the subjects recruited in an acceptable way? |
| 4 | Were the measures accurately measured to reduce bias? |
| 5 | Were the data collected in a way that addressed the research issue? |
| 6 | Did the study have enough participants to minimize the play of chance? |
| 7 | How are the results presented and what is the main result? |
| 8 | Was the data analysis sufficiently rigorous? |
| 9 | Is there a clear statement of findings? |
| 10 | Can the results be applied to the local population? |
| 11 | How valuable is the research? |

| **Author** | **Year** | **Response Rate** | **Sampling technique** | **Screening questions** | | | | | | | | | | |
| --- | --- | --- | --- | --- | --- | --- | --- | --- | --- | --- | --- | --- | --- | --- |
|  |  |  |  | **1** | **2** | **3** | **4** | **5** | **6** | **7** | **8** | **9** | **10** | **11** |
| **Abdelgadir** | 2006 | - | consecutive sampling | + | + | +/- | + | + | + | + | + | + | +/- | + |
| **Adeniyi** | 2015 | - | purposive sampling | + | + | + | + | + | - | + | + | + | +/- | + |
| **Adibe** | 2011 | 78.50% | consecutive sampling | + | + | + | + | + | + | + | + | + | + | + |
| **Adisa** | 2009 | 100% | consecutive sampling | + | + | + | + | + | + | + | + | + | + | + |
| **Adisa** | 2011 | 81.40% | convenient sampling | + | + | + | + | + | + | + | + | + | +/- | + |
| **Awah** | 2008 | - | purposive sampling | + | + | + | + | + | - | + | + | + | +/- | + |
| **Awah** | 2009 | - | purposive sampling | + | + | +/- | + | + | - | + | + | + | +/- | + |
| **Awotibede** | 2016 | - | purposive sampling | + | + | + | + | + | + | + | + | + | + | + |
| **Bruce** | 2015 | 89.30% | consecutive sampling | + | + | + | + | + | + | + | + | + | + | + |
| **de-Graft Aikins** | 2014 | - | consecutive sampling | + | + | +/- | + | +/- | - | + | + | - | +/- | + |
| **Doherty** | 2014 | - | convenient sampling | + | + | + | + | + | + | + | + | + | + | + |
| **Ezuruike** | 2016 | - | convenient sampling | + | + | +/- | + | + | + | + | + | + | +/- | + |
| **Haque** | 2005 | - | consecutive sampling | + | + | + | + | + | - | + | + | + | +/- | + |
| **Hijelm** | 2010 | - | consecutive sampling | + | + | + | + | + | + | + | + | + | + | + |
| **Hijelm** | 2008 | - | consecutive sampling | + | + | + | + | + | + | + | + | + | + | + |
| **Iwuala** | 2015 | - | convenient sampling | + | + | + | + | + | + | + | + | + | + | + |
| **Jackson** | 2014 | 79.70% | consecutive sampling | + | + | + | + | + | + | + | + | + | + | + |
| **Jackson** | 2015 | - | consecutive sampling | + | + | + | + | + | + | + | + | + | + | + |
| **Kamuhabwa** | 2014 | 94.40% | consecutive sampling | + | + | + | + | + | + | + | + | + | + | + |
| **Kassahun** | 2016 | - | - | + | + | +/- | + | + | + | + | + | + | + | + |
| **Matheka** | 2013 | 100% | convenient sampling | + | + | +/- | - | + | +/- | + | + | + | +/- | + |
| **Matwa** | 2003 | - | purposive sampling | + | + | +/- | - | + | - | - | + | - | +/- | + |
| **Mayega** | 2014 | - | purposive sampling | + | + | + | + | + | + | + | + | + | + | + |
| **Mendenhall** | 2015 |  | convenient sampling | + | + | +/- | + | + | - | + | + | + | +/- | + |
| **Mogre** | 2016 | - | convenient sampling | + | + | +/- | - | + | + | + | + | + | + | + |
| **Nielsen** | 2016 | - | convenient sampling | + | + | - | - | + | - | - | + | + | + | + |
| **Nthangeni** | 2002 | - | convenient sampling | + | + | + | + | + | + | + | + | + | +/- | + |
| **Obirikorang_BMC** | 2016 | - | purposive sampling | + | + | + | + | + | + | + | + | + | +/- | + |
| **Obirikorang** | 2016 | - | convenient sample | + | + | - | - | + | + | + | + | + | + | + |
| **Ogbera** | 2011 | - | consecutive sampling | + | + | + | - | + | + | + | + | + | +/- | + |
| **Okonta** | 2014 | - | consecutive sampling | + | + | + | + | + | +/- | + | + | + | +/- | + |
| **Onakpoya** | 2010 | - | consecutive sampling | + | + | + | + | + | - | + | + | + | +/- | + |
| **Oyetunde** | 2014 | 79% | consecutive sampling | + | + | - | + | + | + | + | + | + | - | + |
| **Sorato** | 2016 | - | consecutive sampling | + | + | + | + | + | + | + | + | + | + | + |
| **Steyl** | 2014 | 54.20% | consecutive sampling | + | + | + | + | + | - | + | + | + | +/- | + |
| **Wabe** | 2011 | 90.45 | consecutive sampling | + | + | + | + | + | + | + | + | + | + | + |
| **Yusuff** | 2008 | - | consecutive sampling | + | + | + | + | + | + | + | + | + | + | + |

*(+): low risk of bias; (+/-): unclear risk of bias; (-): high risk of bias*
