# Supplementary material for: Self-management of diabetes in Sub-Saharan Africa: a systematic review
Source: BMC Public Health. 2018 Sep 29;18:1148. doi: 10.1186/s12889-018-6050-0 (PMC6162903; doi:10.1186/s12889-018-6050-0)
Supplement: Supplementary file 4 — Risk assessment for pre-post studies. (DOCX 14 kb) [file 12889_2018_6050_MOESM4_ESM.docx]

**Quality assessment of pre-post, quasi-experimental studies**

Adapted from US Department of Health & Human Services, & National Heart, Lung and Blood Institute. (2014). *Quality Assessment Tool for Before-After (Pre-Post) Studies With No Control Group*

| **#** | **Screening Questions** |
| --- | --- |
| 1 | Was the study question or objective clearly stated? |
| 2 | Were eligibility/selection criteria for the study population prespecified and clearly described? |
| 3 | Were the participants in the study representative of those who would be eligible for the test/service/intervention in the general or clinical population of interest? |
| 4 | Were all eligible participants that met the prespecified entry criteria enrolled? |
| 5 | Was the sample size sufficiently large to provide confidence in the findings? |
| 6 | Was the test/service/intervention clearly described and delivered consistently across the study population? |
| 7 | Were the outcome measures prespecified, clearly defined, valid, reliable, and assessed consistently across all study participants? |
| 8 | Were the people assessing the outcomes blinded to the participants' exposures/interventions? |
| 9 | Was the loss to follow-up after baseline 20% or less? Were those lost to follow-up accounted for in the analysis? |
| 10 | Did the statistical methods examine changes in outcome measures from before to after the intervention? Were statistical tests done that provided p values for the pre-to-post changes? |
| 11 | Were outcome measures of interest taken multiple times before the intervention and multiple times after the intervention (i.e., did they use an interrupted time-series design)? |
| 12 | If the intervention was conducted at a group level (e.g., a whole hospital, a community, etc.) did the statistical analysis take into account the use of individual-level data to determine effects at the group level? |

| **Author** | **year** | **Response Rate** | **Sampling Technique** | **Screening questions** | | | | | | | | | | | |
| --- | --- | --- | --- | --- | --- | --- | --- | --- | --- | --- | --- | --- | --- | --- | --- |
|  |  |  |  | **1** | **2** | **3** | **4** | **5** | **6** | **7** | **8** | **9** | **10** | **11** | **12** |
| Bauman | 2015 | - | convenient sampling | + | + | +/- | +/- | + | + | + | - | +/- | + | - | - |
| Awodele | 2015 | - | consecutive sampling | + | + | +/- | +/- | + | - | + | - | +/- | + | - | - |

*(+): low risk of bias; (+/-): unclear risk of bias; (-): high risk of bias*
