# Supplementary material for: Self-management of diabetes in Sub-Saharan Africa: a systematic review
Source: BMC Public Health. 2018 Sep 29;18:1148. doi: 10.1186/s12889-018-6050-0 (PMC6162903; doi:10.1186/s12889-018-6050-0)
Supplement: Supplementary file 5 — Risk assessment for RCTs. (DOCX 13 kb) [file 12889_2018_6050_MOESM5_ESM.docx]

**Quality assessment of Randomized Controlled Trials**

Adapted from *Reeves BC, Deeks JJ, Higgins JP. 13 Including non-randomized studies. Cochrane Handb Syst Rev Interv. 2008;1:391.*

| **Author** | **Year** | **Response Rate** | **Sampling technique** | **sequence generation** | **allocation sequence concealment** | **blinding of participants and personal** | **incomplete outcome data** | **selective outcome reporting** | **other bias** |
| --- | --- | --- | --- | --- | --- | --- | --- | --- | --- |
| Mash | 2014 | - | consecutive sampling | **+** | + | +/- | + | + | +/- |
| Muchiri | 2015 and 2016 | - | convenient sampling | + | + | +/- | + | + | + |
| Van de doest | 2013 | - | snowball sampling | + | + | +/- | + | + | + |

*(+): low risk of bias; (+/-): unclear risk of bias; (-): high risk of bias*
